# Assembly of Na_3_V_2_(PO_4_)_2_F_3_@C nanoparticles in reduced graphene oxide enabling superior Na^+^ storage for symmetric sodium batteries†

**DOI:** 10.1039/c7ra13441j

**Published:** 2018-01-15

**Authors:** Ye Yao, Lu Zhang, Yu Gao, Gang Chen, Chunzhong Wang, Fei Du

**Affiliations:** Key Laboratory of Physics and Technology for Advanced Batteries (Ministry of Education) State Key Laboratory of Superhard Materials, College of Physics, Jilin University China dufei@jlu.edu.cn gaoyu@jlu.edu.cn

## Abstract

Reduced graphene oxide (rGO) was used to encapsulate Na_3_V_2_(PO_4_)_2_F_3_@Carbon nanoparticles to overcome its inherent low electronic conductivity and achieve superior sodium storage performance. This as-prepared cathode delivers a remarkable rate performance with a discharge capacity of *ca.* 64 mA h g^−1^ at 70C and an ultra-long-term cyclability over 4000 cycles with great capacity retention of 81% at 30C. This excellent performance can be attributed to the favorable combination of fast ionic conductivity of the NASICON structure and the interpenetrating conductive carbon framework; thus bringing a good pseudocapacitive quality to this material. Furthermore, thanks to the good sodium storage properties at low potential, a symmetric full cell can be assembled using Na_3_V_2_(PO_4_)_2_F_3_@C@rGO as both cathode and anode. The full cell delivers a high discharge capacity of 53 mA h g^−1^ at 20C rate, further demonstrating the feasibility of this hybrid material for smart grids.

## Abstract 2

Symmetrical full cell of NVPF@C@rGO as both cathode and anode could light the LED lamp successfully after the 25th charge–discharge cycle at 1C.

## Graphical abstract

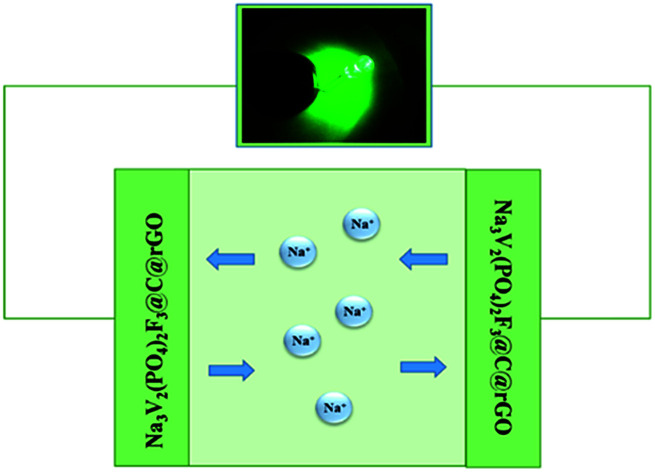

## Introduction

In recent years, sodium-ion batteries (SIBs) have gained worldwide interest as one of the most promising alternatives to lithium-ion batteries (LIBs) owing to their unique merits, including abundant resources, low cost of sodium-containing materials and an identical working principle to LIBs.^1–6^ However, much effort still should be devoted to the exploration of novel electrode materials because the larger ionic radius of Na^+^ usually induces sluggish kinetics during the electrochemical insertion reactions with unsatisfactory rate capability and cycle stability.^7–12^

NASICON-type (Na^+^ super-ionic conductor) phosphates are characteristics as the robust three-dimensional open frameworks facilitating fast ionic transportation.^8,13^ Among various NASICON type materials, sodium–vanadium fluorophosphates, Na_3_V_2_(PO_4_)_2_F_3_ attracts particular attention because of the high theoretical energy density of 475 W h kg^−1^, approaching that of LiFePO_4_ (530 W h kg^−1^) in LIBs.^14–18^ Unfortunately, its low electronic conductivity of 10–12 S cm^−1^ limits the synergic transfer between the electrons and ions; thus the high theoretical capacity can hardly be achieved.^18,19^ To improve the sodium storage performance, the popular strategy is the preparation of nanocomposite materials with various carbonaceous materials, like CNT, amorphous carbon, and *etc.*^20–23^ On one hand, carbonaceous materials can help to reduce the particle size and shorten the ionic transfer pathway;^19,24^ on the other, the high conductivity of working electrode is greatly improved, facilitating fast electronic transportation.^23,25^ Besides the abovementioned methods, pseudocapacitive charge storage, which is governed by surface or near-surface reversible redox reaction, is also another effective way to realize high-rate charge–discharge behavior.^14,26,27^ It has been demonstrated that pseudocapacity can bring in well balance in the energy and power density of the electrode materials. Though many nano-sized anode materials, including Nb_2_O_5_,^28^ RuO_2_ (ref. 29) and *etc.* has exhibited enhanced pseudocapacitive contribution, there is a great challenge to exploit the intercalation pseudocapacity in the positive side.

In this work, we synthesized NVPF nanoparticles embedded in the reduced graphene oxide (rGO) by a nanocasting technique to realize a high-rate charge–discharge ability.^25,30^ Thanks to the improved electronic conductivity and enhanced pseudocapacitive charge storage, the NVPF@C@rGO hybrid electrode demonstrated a superior rate capability with a discharge capacity of 64 mA h g^−1^ at 70C rate and ultralong cycle life over 4000 cycles with a capacity retention of 81% at 30C rate. In addition, a symmetric cell based on NVPF@C@rGO as both cathode and anode was assembled successfully, which showed a high reversible capacity of 107 mA h g^−1^ at 1C and 53 mA h g^−1^ at 20C indicative of the promising application in the grid energy storage.

## Results and discussion

**Fig. 1:**
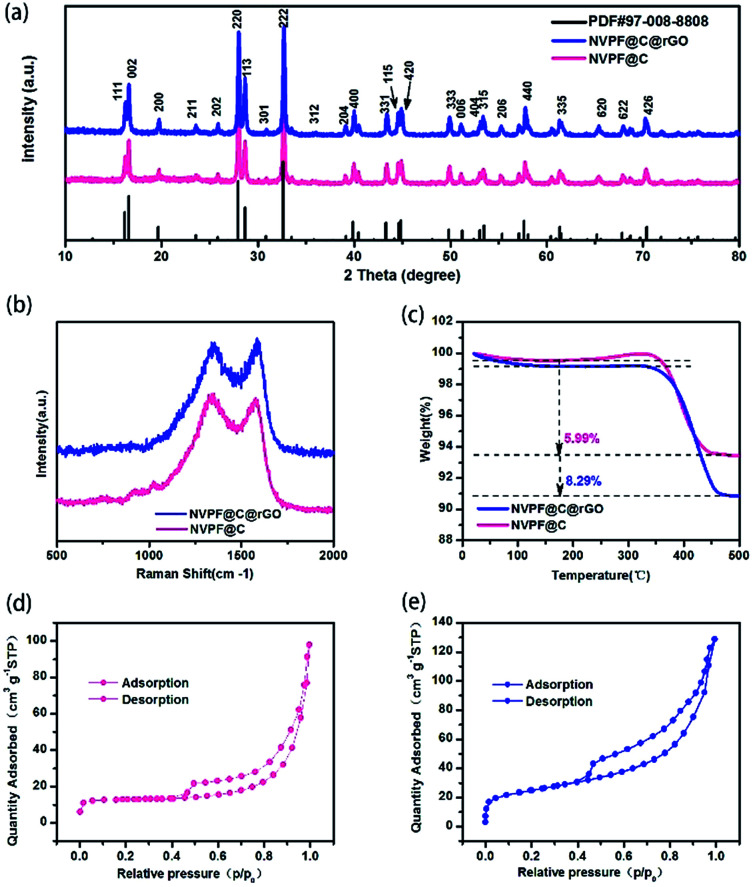
X-ray diffraction pattern of the pristine NVPF@C@rGO and NVPF@C nanocomposites (a); Raman spectrum of NVPF@C@rGO (b); thermogravimetric measurement (TG) profile of the pristine NVPF@C@rGO and NVPF@C nanocomposites(c); nitrogen adsorption–desorption isotherms of NVPF@C (d) and NVPF@C@rGO (e).

The XRD patterns of NVPF@C@rGO and NVPF@C are compared in Fig. 1a, with no observed second phases. All the diffraction peaks can be indexed based on the tetragonal symmetry with the space group of *P*42/*mnm* and the lattice parameters are calculated and listed in Table S1.† The introduced carbon was further confirmed by Raman spectroscopy as displayed in Fig. 1b. Two typical Raman peaks are identified at ∼1342 and ∼1588 cm^−1^, corresponding to D and G bands, respectively. The relative intensity ratio (*I*_D_/*I*_G_) is usually used as a criterion to evaluate the degree of disorder and defects in carbonaceous materials.^31^ After introducing rGO, the *I*_D_/*I*_G_ decreased from 1.027 (NVPF@C) to 0.960 (NVPF@C@rGO), suggesting the lower degree of disorder and a better crystallinity of carbon. Quantitatively, TG measurement was used to estimate the content of carbon in the pristine nanocomposite. As shown in Fig. 1c, the contents of carbon for the NVPF@C and NVPF@C@rGO samples were *ca.* 5.99 wt% and 8.29 wt%, respectively. Therefore, the content of rGO is 2.3%. The nitrogen adsorption–desorption isothermal technique was further employed to examine detailed micro-structural information. After compositing with rGO (Fig. 1d and e), NVPF@C@rGO hybrid exhibits a larger BET surface are a (86 m^2^ g^−1^) in compare with the value of NVPF@C (41 m^2^ g^−1^), because of the layer characteristic of rGO. Higher BET surface area might increase a larger electrolyte–electrode contact area for better sodium storage performance.

**Fig. 2:**
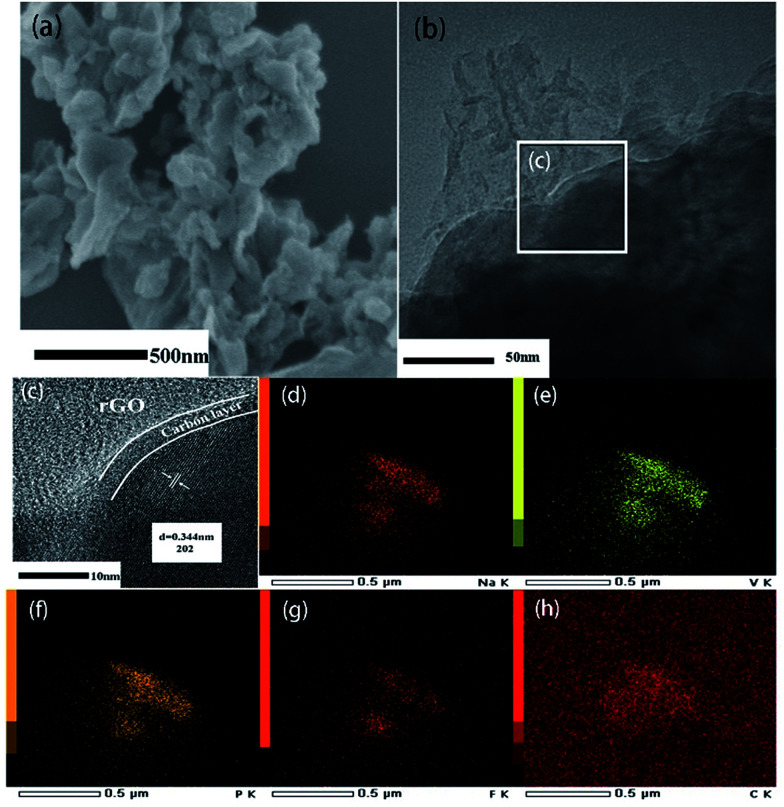
SEM (a), TEM (b) and HRTEM (c) images of NVPF@C @rGO; (d)–(h) represents the HAADF-STEM image and elemental mapping of Na, V, P, F and C, respectively.

Morphological features of two samples were observed by SEM and TEM. It can be seen that NVPF nanoparticles with tens to hundreds of nanometers in size are wrapped by the flexible rGO nanosheets (Fig. 2a). TEM image (Fig. 2b and c) not only confirms the morphologic features of SEM, but also indicates a thin carbon layer (3–4 nm) is coated on the surface of NVPF particles. This hierarchical carbon coating structure is helpful to limit the growth of NVPF particles and act as an effective electronic transfer network. HRTEM images (Fig. 2c) clearly suggest a lattice fringes of 0.344 nm, corresponding to 202 plane of tetragonal NVPF. In contrast, NVPF@C displays an agglomerated nanoparticles wrapped in the amorphous carbon matrix with non-uniformly dispersed particle size ranging from 200 to 500 nm, as displayed in Fig. S1.† In addition, the element mappings (Fig. 2d–h) demonstrated a uniform distribution of various elements (Na, V, P, F and C) in the NVPF@C@rGO nanocomposite.

**Fig. 3:**
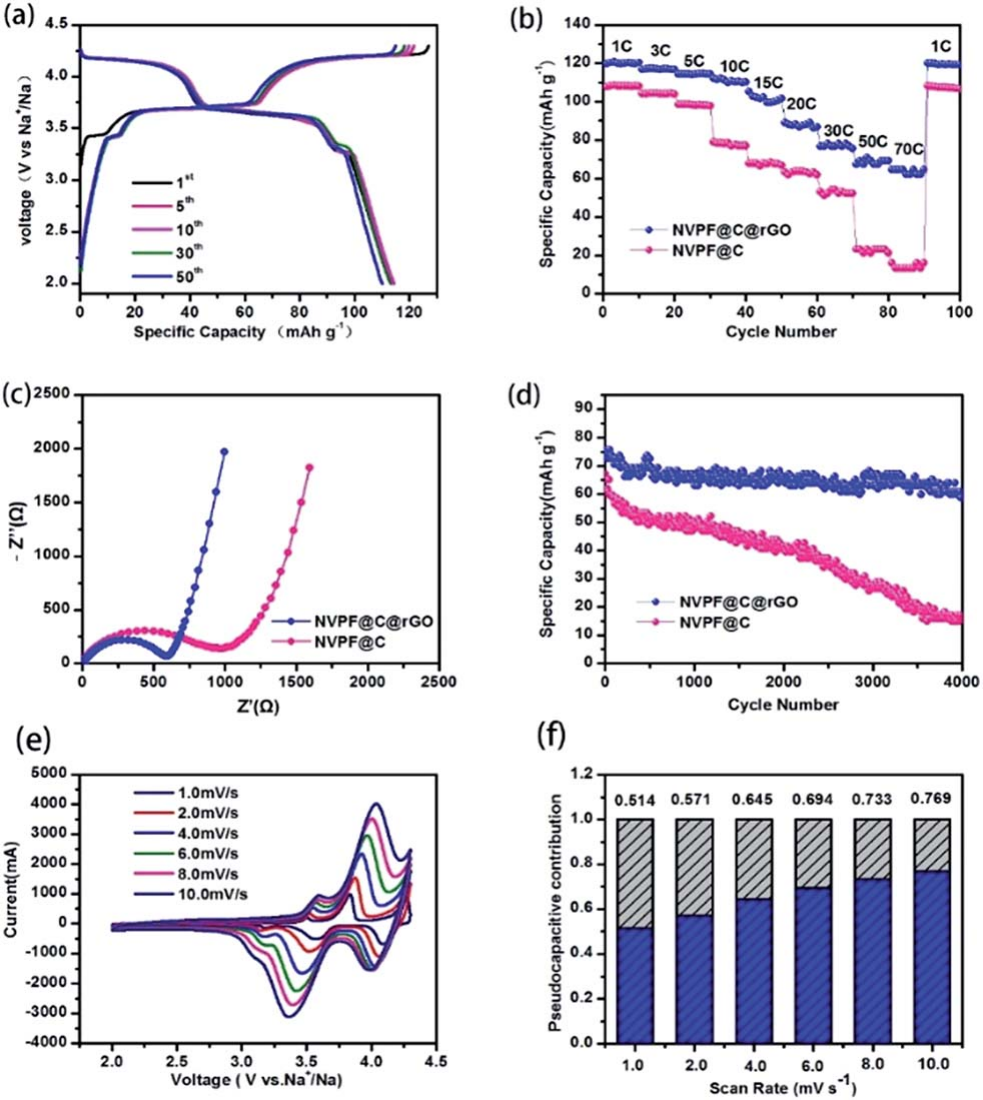
Charge–discharge profiles of NVPF@C@rGO at 0.5C rate (a); rate performances at various current densities (b), the Nyquist plots after the 50th cycles (c) of NVPF@C@rGO and NVPF@C; long-term cycle stability of the NVPF@C@rGO and NVPF@C tested at 30C rate for 4000 cycles (d). CV curves of NVPF@C@rGO at sweep rates from 1 to 10 mV s^−1^ (e); diagram of capacitive contribution to the total capacity (f).

The electrochemical properties of NVPF@C@rGO and NVPF@C were evaluated using coin-type cells with sodium metal as the counter electrode. Fig. 3a shows the represented galvanostatic charge–discharge profiles of NVPF@C@rGO between 2.0 and 4.3 V at a current density of 0.5C rate, consistent with those profiles of NVPF@C (Fig. S2†). The initial charge capacity is obtained as *ca.* 127 mA h g^−1^, close to the theoretical capacity of NVPF (128 mA h g^−1^) when two Na^+^ ions are extracted. Since the carbonaceous materials, including amorphous carbon and rGO, are only capable of accommodating sodium ions at low potential (below 1.0 V) and their capacity contribution in the high voltage region of above 2.0 V is negligible.^32^ Furthermore, NVPF@C@rGO demonstrates superior rate capability. As shown in Fig. 3b, the cathode delivers reversible capacities of 120, 116, 114, 112, 105, 89, 77, 71, and 64 mA h g^−1^ at current densities of 1, 3, 5, 10, 15, 20, 30, 50 and 70C, respectively. Particularly, when the current density returns to 1C after 100 cycles, the specific capacity is nearly recovered to the same extent as its initial ten cycles, indicative of an excellent reversibility even at high-rate charge–discharge. In contrast, the NVPF@C nanocomposite can sonly deliver 16 mA h g^−1^ at 70C rate, much lower than that of NVPF@C@rGO. To elucidate the reason for the enhanced rate performance after compositing with rGO, EIS is recorded and displayed in Fig. 3c. Both EIS curves are composed of a semicircle in the high frequency and medium frequency region.

Characteristic of the charge-transfer reaction and one straight line in the low frequency region reflecting the sodium-diffusion process in the bulk phase.^33,34^ After quantitative fitting of the impedance spectra using the equivalent circuit (Fig. S3†), the charge-transfer resistance (*R*_ct_) of NVPF@C@rGO cell are calculated to be 561.1 Ω, lower than that value of NVPF@C (811.2 Ω) at the same electrochemical stages. The decrease in the *R*_ct_ might be related to the hierarchical architecture design with carbon coated NVPF wrapped in rGO nanosheets; thus accelerating electronic and ionic transfer of the working electrode.^35^ The inclined line in the low frequency region represents the Warburg impedance, which is associated with the Na^+^ ion diffusion in the NVPF particles. The apparent diffusion coefficient of Na^+^, *D*_Na_, can be calculated by the equation^36^1
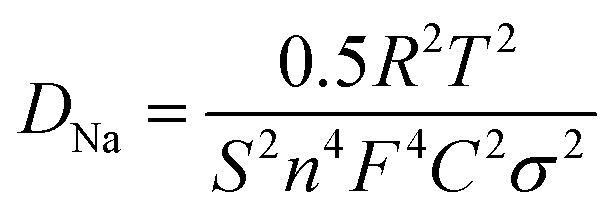


In this equation, *R* is the gas constant, *T* the absolute temperature, *F* the Faraday constant; and *σ* is the Warburg factor which obeys the following relationship:

2 *Z*′ = *R*_S_ + *R*_CT_ + *σω*^−1/2^

Fig. S4† displays the linear fitting of Z′ *vs. ω*^−1/2^, from which the slope *σ* can be obtained. The sodium diffusion coefficients of NVPF@C and NVPF@C@rGO are 1.32 × 10^−13^ and 3.08 × 10^−12^, respectively. The slightly improved diffusion coefficient maybe because the increase of conductivity, which related to rGO. Inspired by the outstanding rate performance, the long-term cycle stability of NVPF@C@rGO and NVPF@C was also examined (Fig. 3d). The hybrid electrode delivers a discharge capacity of 60.8 mA h g^−1^ after 4000 cycles at 30C, corresponding to 81% of its initial capacity. In sharp contrast, NVPF@C could only deliver a low discharge capacity of 14.9 mA h g^−1^ after 4000 cycles.

To further understand the superior rate capability of NVPF@C@rGO hybrid electrode, the kinetic properties are investigated by cyclic voltammetry (CV) at various scanning rates. Fig. 3e shows the CV curves at sweep rates of 1 mV s^−1^ to 10 mV s^−1^. There are three redox couples corresponding to the three symmetrical working plateaus observed at *ca.* 3.3, 3.6 and 4.1 V in both charge and discharge profiles. The first two redox couples can be attributed to a two-step process insertion into/extraction of Na^+^ from Na(2) sites, while the higher potential at around 4.2 V is derive from the Na(1) sites.^19,37^ The redox peaks deviated from the equilibrium position due to the larger polarization under the higher sweeping speed. Relationships between the peak currents and scan rates in logarithmic format indicated the type of electrochemical behaviour. Generally, the slope of 0.5 indicates a diffusion-controlled behaviour, whereas the 1.0 represents a surface diffusion process, the slope values of second pairs of peaks were calculated to 0.61 and 0.79, indicating a pseudocapacitive process between typical behaviours of batteries and capacitors.^38–40^ The total capacitive contribution at a certain scan rate could be quantified by separating as combination of surface capacitive effects and diffusion-controlled process according to^39^

3 *i*(*V*) = *k*_1_*V* + *k*_2_*V*^1/2^

where *k*_1_ and *k*_2_ are constants for a given potential. Quantitative calculation results (Fig. 3f) reveal that the value of capacitive contribution at scan rate of 1 mV s^−1^ is 51%, and gradually increases as the scan rate increases, finally reached to 77% at 10 mV s^−1^. All redox peaks are broadened at high sweep rates, which conforms the capacitive behaviour at high rates.

**Fig. 4:**
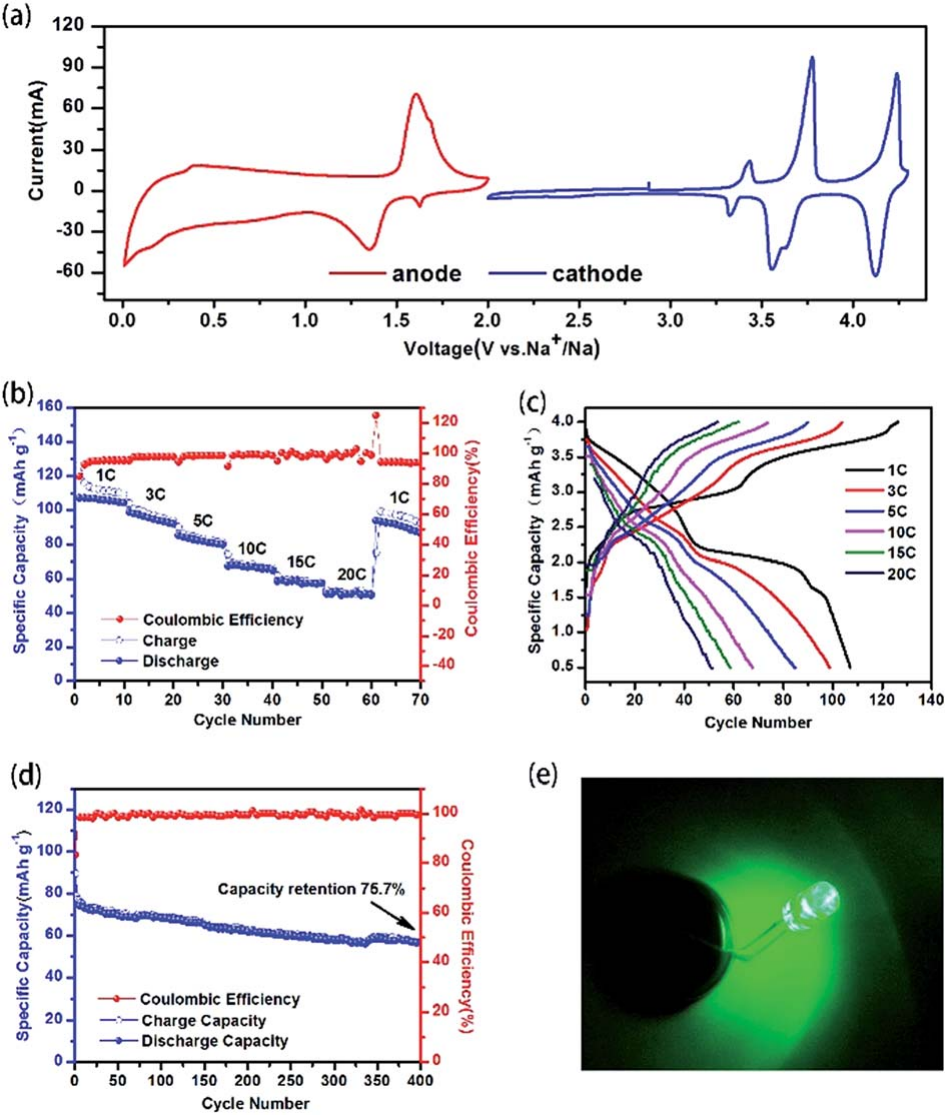
Electrochemical properties of NVPF@C@rGO as symmetrical full cell material. Cyclic voltammetry curves of NVPF@C@rGO as cathode and anode (a); rate performances (b) and charge–discharge profiles at various current densities (c) of the NVPF@C@rGO symmetrical full cell; the cycling performance of the NVPF@C@rGO symmetrical full cell at 10C (d); the lighted LED bulbs driven by the full cell (e).

Moreover, a symmetric SIBs full cell is assembled configured [Na_3_V_2_(PO_4_)_2_F_3_@C@rGO‖1 M NaClO_4_ in (EC : PC = 1 : 1)‖Na_3_V_2_(PO_4_)_2_F_3_@C@rGO]. Before investigating the electrochemical performance of the full cell, the CV profiles (Fig. 4a) of NVPF@C@rGO in both cathode and anode are evaluated. NVPF@C@rGO demonstrates a working plateau at *ca.* 1.4 V *vs.* Na^+^/Na when being discharged to 0.01 V with discharge capacity of 95 mA h g^−1^ (Fig. S5†). The mass balance between the cathode and anode is fixed as 1 : 1.3 to ensure the full activation of NVPF during the initial charging process and improve the initial coulombic efficiency of the symmetric cell. Fig. 4b evaluates the rate capability for every ten successive cycle. A high discharge capacity of 107 mA h g^−1^ is delivered at 1C rate and 53 mA h g^−1^ for 20C. As increased in the C rate, the working plateaus in the charge–discharge profiles disappears gradually owing to the enhanced polarization (Fig. 4c). The cycle stability are also recorded for 400 cycles at Fig. 4d with capacity retention of 75.7% at 10C. Furthermore, LED lamp could be lit successfully by this full cell after 25th charge–discharge cycle at 1C (Fig. S6† and 4e), suggesting great practical prospect.

## Experimental

### Synthetic procedures

The main paragraph text follows directly on here. Graphene oxide (GO) was prepared according to Hummers method.^41^ To prepare the NVPF@C@rGO nanocomposite, 0.2 mmol NH_4_VO_3_, 0.3 mmol NaF, and 0.2 mmol NH_4_H_2_PO_4_ powders were first dissolved in deionized water. Citric acid (0.29 g) was then added and constantly stirred at room temperature for 12 hours. Second, 0.042 g GO and 42 ml deionized water were ultrasonically treated for 2 hours to form a uniform dispersion, and then added into the above solution with stirring at room temperature for another 12 hours. Third, the mixed solution was quick freezed with liquid nitrogen then freeze-drying at −40 °C for 48 h to slowly evaporate the solvent. Finally, the precursor powder sample was acquired after a two-step heat treatment: 300 °C for 4 hours and then 650 °C for 8 hours under a nitrogen atmosphere to crystallize NVPF and reduce GO. In contrast, NVPF@C nanocomposite was prepared following the identical procedure of NVPF@C@rGO without adding rGO.

### Materials and methods

The phase purity of the obtained samples was examined by X-ray diffraction (XRD; RigaKu D/max-2550) with Cu Kα source in the 2*θ* range of 10–80° at a scanning rate of 2° min^−1^. The morphological features were observed by a Hitachi SU8020 scanning electron microscope (SEM) and a FEI Tecnai G2 transmission electron microscope (TEM). Raman spectroscopy was recorded on a Renishaw in *via* Raman spectroscopy with Ar-ion laser excitation (*λ* = 514.5 nm). Quantitatively, the content of carbon was evaluated using a Mettler-Toledo Thermogravimetric (TG) analyzer. Nitrogen adsorption–desorption isotherms were measured at 77 K using a Micromeritics ASAP 2010 instrument. The specific surface area was calculated using the Brunauer–Emmett–Teller (BET) method and the pore-size distribution (PSD) curves were calculated from the isotherm using the Barrett–Joyner–Halenda (BJH) algorithm.

### Electrochemical measurements

Electrochemical experiments were carried out using 2032-type coin cells. A typical electrode was composed of 70 wt% active material, 20 wt% super P conductive additive, and 10 wt% polyvinylidene fluoride binder (PVDF) dissolved in *N*-methylpyrrolidone (NMP). The obtained slurry mixture was pasted onto aluminium foil and then dried at 120 °C for 12 h in a vacuum oven. After dividing the electrode film into square parts of 0.8 × 0.8 cm^2^, coin cells were assembled in a glove box (O_2_ ≤ 0.1 ppm, H_2_O ≤ 0.1 ppm). The loading mass of the active material in each coin cell is typically 1.2–1.5 mg cm^−2^. The cathode and anode electrodes were separated by a glass fiber filter (Whatman GF/C). The electrolyte was 1 M NaClO_4_ dissolved in a solvent of ethylene carbonate (EC) and propylene carbonate (PC) (1 : 1 v/v) and 5% fluoroethylene carbonate (FEC). Galvanostatic charge–discharge tests were carried out at a voltage window of 2.0–4.3 V on a Land-2001A (Wuhan, China) automatic battery tester at room temperature. Here, C rate was used to characterize the current rate; 1C equaled 128 mA g^−1^. The specific capacity was calculated on the basis of the active cathode material after subtracting the carbon content. Data of cyclic voltammetry (CV) and electrochemical impedance spectra (EIS) were recorded on a VSP multichannel potentiostatic–galvanostatic system (Bio-logic, France). The test conditions of EIS were in the frequency range from 1 MHz to 1 mHz with an ac voltage of 5 mV.

## Conclusions

NVPF@C@rGO nanocomposite was successfully synthesized by the nanocasting technique, where carbon-coated NVPF nanoparticles were wrapped by rGO nanosheets. Owing to the enhanced kinetics properties, NVPF@C@rGO hybrid electrode demonstrates a superior sodium storage performance in comparison with NVPF@C material. By analyzing the DSCV, a strong pseudocapacitive contribution is found which well explains the high-rate charge–discharge ability. The symmetrical cell with NVPF@C@rGO as both cathode and anode was, for the first time, constructed, further demonstrating the feasibility of NVPF@C@rGO nanocomposite in SIBs.

## Conflicts of interest

There are no conflicts to declare.

## Supplementary Material

RA-008-C7RA13441J-s001
