# Supplementary material for: Assembly of Na3V2(PO4)2F3@C nanoparticles in reduced graphene oxide enabling superior Na+ storage for symmetric sodium batteries
Source: RSC Adv. 2018 Jan 15;8(6):2958–62. doi: 10.1039/c7ra13441j (PMC9077572; doi:10.1039/c7ra13441j)
Supplement: RA-008-C7RA13441J-s001 [file RA-008-C7RA13441J-s001.pdf]

Electronic Supplementary Material (ESI) for RSC Advances.
This journal is © The Royal Society of Chemistry 2017

Assembly of $Na_3V_2(PO_4)_2F_3$@C Nanoparticles in Reduced Graphene Oxide Enabling Superior Na^+ Storage for Symmetric Sodium Batteries

Ye Yao,[a] Lu Zhang,[a] Yu Gao*[a], Gang Chen,[a] Chunzhong Wang[a] and Fei Du*[a]

Key Laboratory of Physics and Technology for Advanced Batteries (Ministry of Education), State Key Laboratory of Superhard Materials, College of Physics, Jilin University, Changchun, 130012, People's Republic of China

Email: dufei@jlu.edu.cn; gaoyu@jlu.edu.cn

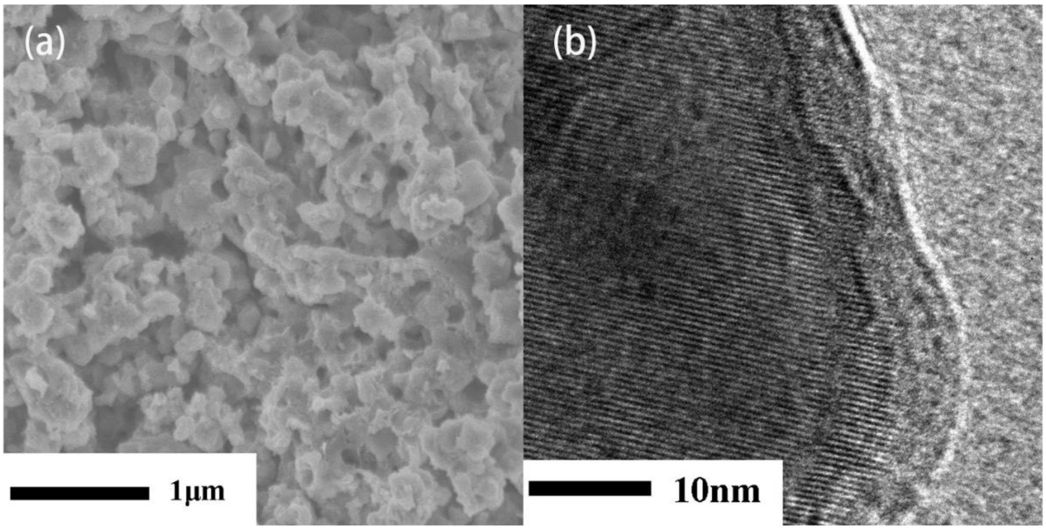

Figure S1. SEM (a)and TEM(b) of NVPF@C

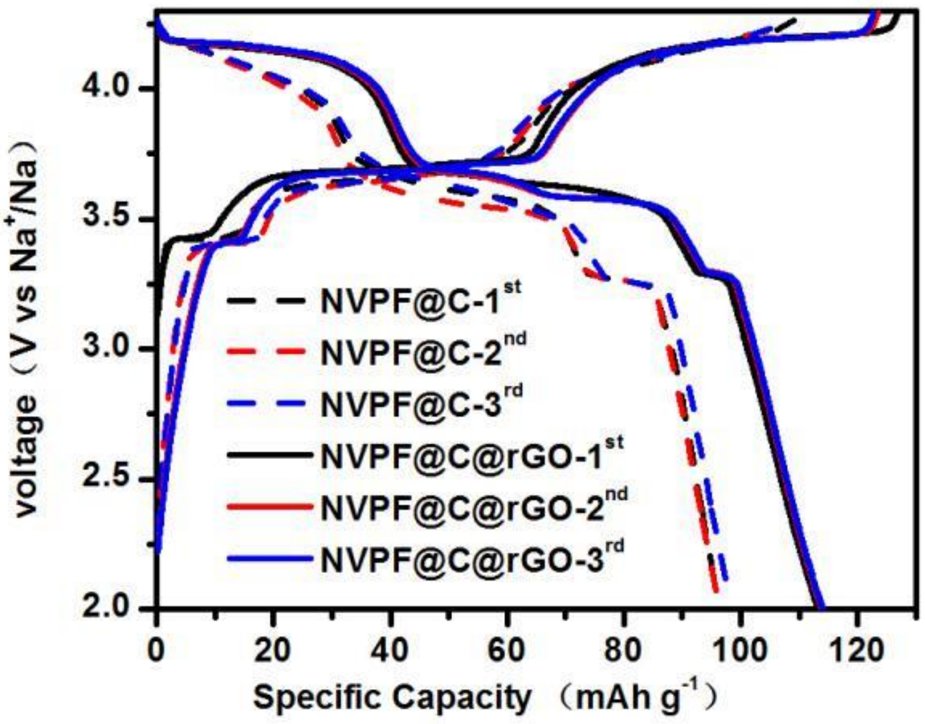

Figure S2. Charge-discharge profiles of the NVPF@C and NVPF@C@rGO.

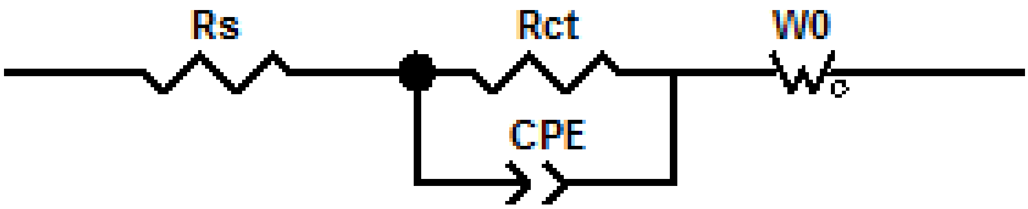

Figure S3. Equivalent circuit for the Nyquist plots of the NVPF@C and NVPF@C@rGO.

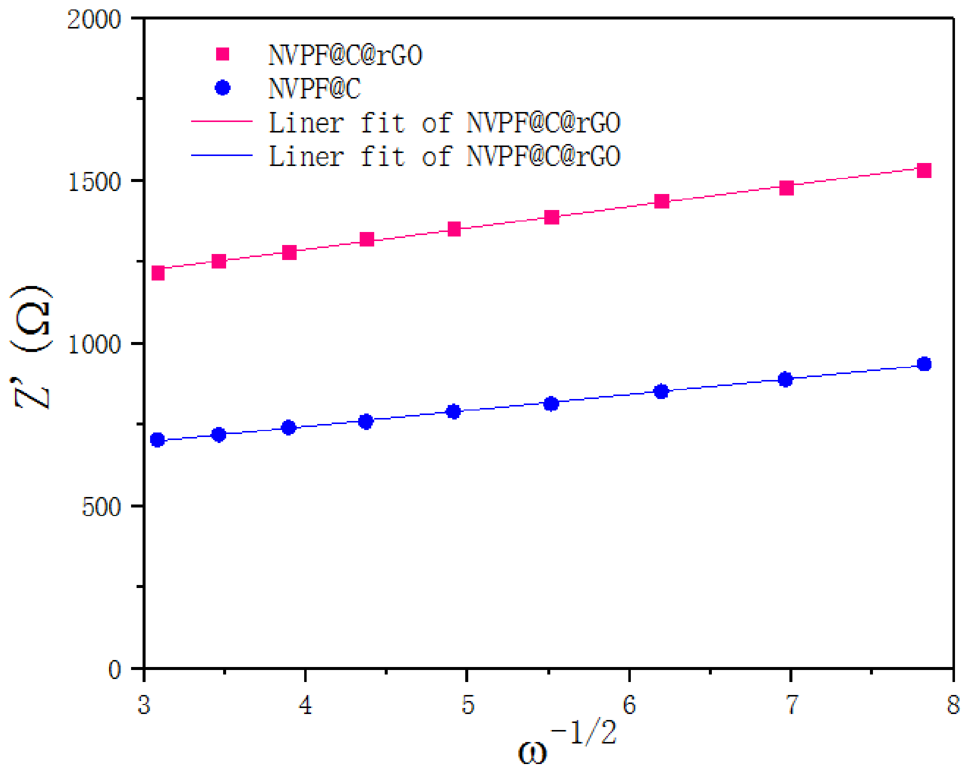

Figure S4. Linear fitting of Z' vs. $\omega^{-1/2}$ of NVPF@C and NVPF@C@rGO.

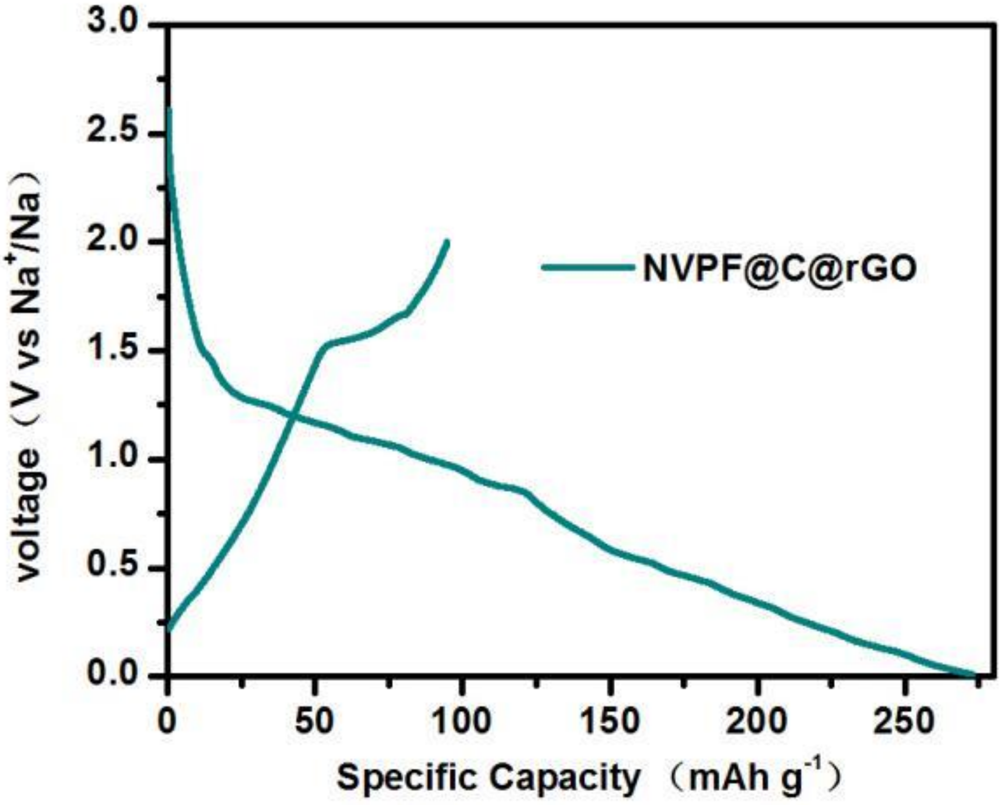

Figure S5. Charge-discharge profile of NVPF@C@rGO in the voltage range of 0.01-2.0V at 1C rate.

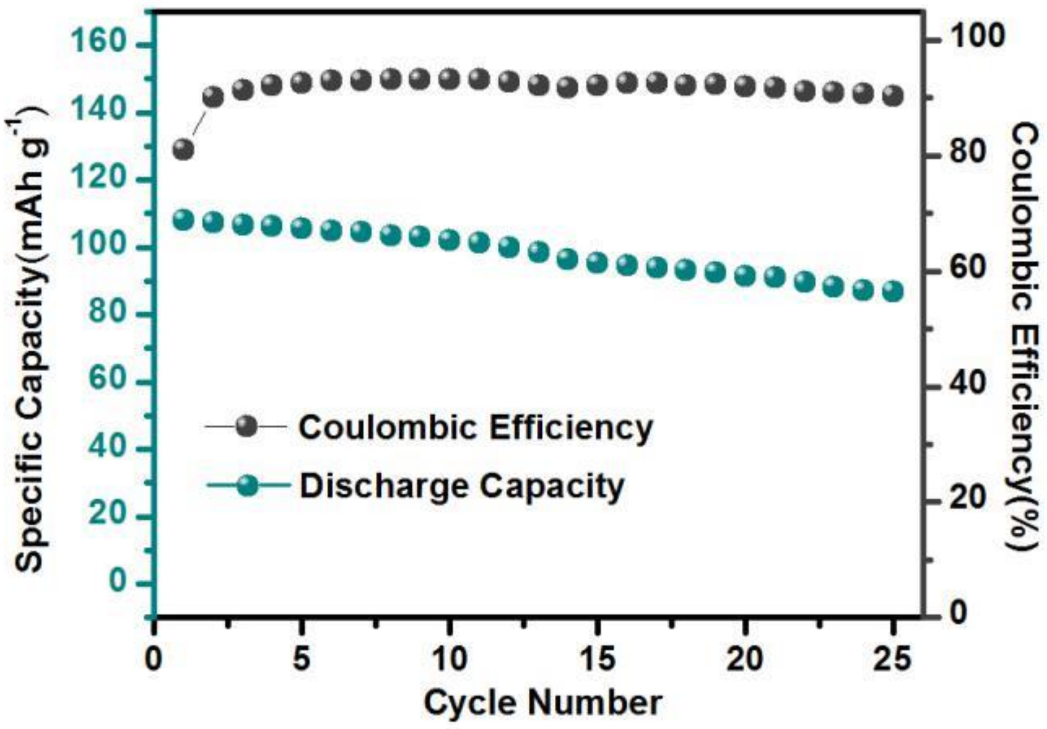

Figure S6. Cycle performance of NVPF@C@rGO symmetric full cell at 1C.
